# The Association Between Cognitive Function and Preventive Care Service Utilization Among U.S. Chinese Older Adults

**DOI:** 10.1093/geroni/igaa057.1180

**Published:** 2020-12-16

**Authors:** Zhing Loong Poh, Dexia Kong, Mengting Li, XinQi Dong

**Affiliations:** 1 Rutgers University, The State University of New Jersey, Chicago, Illinois, United States; 2 Rutgers University, New Brunswick, New Jersey, United States

## Abstract

Preventive healthcare utilization is an important aspect of medical practice that facilitates the identification of chronic diseases at an early stage and increases options for treatment. Cognitive function plays an important role in individuals’ utilization of preventive care services. However, our understanding of the relationship between cognitive function and preventive care utilization is limited, particularly in older minority aging populations. The study aims to assess the association between cognitive function and preventive healthcare utilization among U.S. Chinese older adults. Data were obtained from the Population Study of Chinese Elderly in Chicago (PINE). Five instruments were used to measure global cognition, including the Mini-Mental State Examination, East Boston Memory Test Immediate Recall and Delayed Recall, Digit Span Backwards, and Symbol Digit Modalities Test. Preventive care services included immunization (i.e. flu, pneumonia, and hepatitis B vaccines) and cancer screenings (i.e. colorectal, breast, cervical, and prostate). Multivariable regression analyses were used. The findings showed that higher level of global cognition was associated with higher utilization of pneumonia vaccination (OR=1.32, 95% CI= 1.14-1.52), hepatitis B vaccination (OR=1.24, 95% CI= 1.05-1.47), colon exam (OR=1.23, 95% CI= 1.07-1.41), mammogram (OR=1.46, 95% CI= 1.22-1.73), breast exam (OR=1.23, 95% CI= 1.04-1.46), and cervical exam (OR=1.38, 95% CI= 1.15-1.65). Future longitudinal studies are needed to elucidate potential mechanisms underlying the relationship between cognitive function and preventive care utilization among U.S. Chinese older adults. Study findings underscore the need to understand preventive care utilization patterns among U.S. Chinese older adults with low cognitive function.

